# Status and influencing factors of weight loss behavior among college students: a cross-sectional study

**DOI:** 10.3389/fpubh.2026.1780638

**Published:** 2026-04-15

**Authors:** Ding Xu, Cuixia Lin, Beibei Liang

**Affiliations:** 1 School of Nursing, Shandong University of Traditional Chinese Medicine, Jinan, Shandong, China; 2 School of Health, Shandong University of Traditional Chinese Medicine, Jinan, Shandong, China

**Keywords:** college students, cross-sectional study, influencing factors, obesity, weight loss

## Abstract

**Background:**

College students are the main force of the country’s future. The physical health of college students is directly related to the country’s future development and progress. Overweight and obesity not only affect the appearance of college students, but also increase the risk of illness, and affect their study, life and career development. Therefore, this study aims to evaluate the current status of weight loss behavior and its influencing factors in college students, in order to provide baseline data for the development of targeted weight loss measures, and provide a basis for improving the physical and mental health of college students and long-term weight management goals.

**Methods:**

Using the convenience sampling method, 302 Chinese full-time college students from a traditional medicine university in Shandong Province were selected as the survey subjects from July to August 2025. Questionnaire surveys were conducted using the General Information Questionnaire, the Dutch Eating Behavior Questionnaire, the Chinese version of the Self-Efficacy for Exercise Scale, and the Health Status Survey Scale. SPSS 26.0 software was used to process and analyze the data. Descriptive statistics were used to analyze the general information. Logistic regression was used to analyze the influencing factors of weight loss among college students and conducted a correlation analysis.

**Results:**

45.0% of college students had weight loss behavior, among whom 63 were male and 73 were female. Dutch Eating Behavior, Self-Efficacy for Exercise were positively correlated with weight loss behavior of college students. Logistic regression analysis showed that there were statistically significant differences between body mass index, weight loss intention, Dutch Eating Behavior, Self-Efficacy for Exercise and weight loss behavior of college students, *p* < 0.05.

**Conclusion:**

Body mass index, weight loss intention, Dutch Eating Behavior, and Self-Efficacy for Exercise were the influencing factors of weight loss behavior among college students. Relevant departments of the school should do good job in health education for students, guide them to establish good lifestyle, improve their dietary management skills and confidence in exercise, strengthen their motivation for weight loss, enhance self-health management, and maintain healthy weight.

## 1 Introduction

Obesity is a systemic disease characterized by excessive and abnormal accumulation of fat tissue in the body (1). The prevalence of obesity has been increasing year by year worldwide and has become a global public health issue (2). The World Obesity Atlas 2025 published by the World Obesity Federation has shown that the total number of adults living with obesity will be projected to increase by more than 115% between 2010 and 2030 (3). The number of overweight adults worldwide would reach 2.7 billion and the number of obese adults would exceed 1 billion by 2025 (4). The problem of overweight and obesity in China cannot be ignored either. At present, the obesity rate among adults in China has reached as high as 50%. It has been estimated that the prevalence of obesity among adults in China will reach 65.3% by 2030 (5). With the acceleration of the pace modern life, the change of dietary structure, the normalization of sedentary behavior, and the reduction in physical activity among college students due to isolation after the outbreak of the novel coronavirus disease, the problems of overweight and obesity have become increasingly prominent among college students and have become important factors affecting their physical and mental health (6, 7).

A study (8) surveyed 13,082 college students with an average age of 20 and found that the rate of overweight and obesity among college students was as high as 36.05%. Overweight and obesity not only affect the appearance of college students, but also increase the risk of cardiovascular diseases, type 2 diabetes (9, 10), sleep apnea (11), osteoarthritis (12), metabolic syndrome, non-alcoholic fatty liver disease and a variety of cancers (10). These diseases not only affect the study, life and future career development (9), but also bring pain to college students and increase the burden on the medical system. It has been predicted that the medical expenses of overweight and obesity in China will account for 22% of the total medical expenses in China by 2030 (5). Obesity not only seriously endangers individual’s physical and mental health, reduces the quality of life, but also brings economic burden to society. College students are the main force of the country’s future. The physical health of college students is directly related to the country’s future development and progress. Reducing the incidence of overweight and obesity among college students has become an urgent problem to be solved. Therefore, this study investigated the attitudes, current situation and influencing factors of weight loss among college students, which would be of great significance for formulating effective intervention measures, improving the dietary habits of college students, enhancing their physical fitness and promoting their physical and mental health.

## 2 Materials and methods

### 2.1 Study design and participants

By using the convenience sampling method, a cross-sectional survey was conducted among full-time college students of a traditional Chinese medicine university in Shandong Province from July to August 2025 through classmate sharing, WeChat Moments and group chats. Inclusion criteria: (1) College students currently enrolled; (2) Age ≥ 18 years old; (3) Informed consent and voluntary participation in this study. Exclusion criteria: (1) The response time was less than 120 s; (2) Regular responses or logical abnormalities. The purpose and significance of the study were explained to the subjects before the study, and the investigation was conducted after obtaining the informed consent of the subjects.

### 2.2 Measures

#### 2.2.1 Questionnaire survey

The general information questionnaire mainly included gender, age, height, weight, residence, education level, grade, weight loss intention, and parents’ education level.

The Dutch Eating Behavior Questionnaire (DEBQ) was used to assess patients’ eating problems and predict overweight, obesity and possible eating disorders. This scale was compiled by VanStrien et al. (13) in 1986, with a total of 33 items, including three dimensions of restrained eating, emotional eating and external eating, using the 5-level Likert scoring method. Domestic scholars Li et al. (14) translated the DEBQ into Chinese and revised it among domestic college students. The Cronbach’s *α* coefficient was 0.88, and the validity was 0.91. The higher the score, the more severe the individual’s eating problem. In this study, the Cronbach’s *α* coefficient of this scale was 0.951 and its validity was 0.948.

The Chinese version of the Self-Efficacy for Exercise Scale (SEE-C) was used in clinical practice to measure and evaluate individual’s confidence level in persisting in exercise when facing various obstacles. The Self-Efficacy for Exercise Scale (SEE) was developed by Resnick et al. (15). Taiwanese scholars Lee et al. (16) localized and culturally adapted SEE to form SEE-C. Subsequently, Zhang et al. (17) applied SEE-C to the obese population, and the Cronbach’s *α* coefficient was 0.878. SEE-C consisted of 1 dimension and 9 items. The conditions described in each item would affect regular exercise three or more times a week. The subjects scored each item based on their confidence level in performing regular exercise, ranging from 0 to 10 points according to the degree of confidence, from no confidence at all to very confident. The total score ranged from 0 to 90 points. The higher the score, the higher the self-efficacy in sports. In this study, the Cronbach’s *α* coefficient of this scale was 0.949 and its validity was 0.929.

36-item short-form health survey (SF-36) was a commonly used health-related quality of life assessment tool. It was developed by the American Institute of Medicine to assess individual’s health condition and quality of life. The SF-36 included assessment of individual’s physical and mental health status, with 36 questions covering 8 dimensions: physical function (PF), role physical (RP), bodily pain (BP), general health (GH), vitality (VT), social function (SF), role emotion (RE), mental health (MH). Through the assessment of these aspects, SF-36 could help medical professionals and researchers understand individual’s overall health status and guide clinical decision-making and health research. Due to its universality and effectiveness, SF-36 has been translated into multiple languages and widely used around the world. This study adopted the Chinese version translated by Professor Fang (18). The Cronbach’s *α* coefficient was 0.824. The higher the score, the better the patient’s health condition and the higher the quality of life related to health. In this study, the Cronbach’s *α* coefficient of this scale was 0.817 and its validity was 0.832.

The critical attributes for weight management included dietary measures, physical activity, behavior modification, motivation, education, and lifelong changes. Successful weight management practices included the prevention of weight gain, weight loss, and maintenance of ideal body weight (19). The operational definition of weight loss behavior in this study referred to the current actions taken by the respondents to reduce weight, which involved specific actions such as dietary control (reducing intake of high-calorie foods, controlling food intake, etc.), increasing physical activity (regular exercise, increasing daily activity volume, etc.), and adjusting lifestyle (avoiding staying up late, reducing prolonged sitting, etc.).

#### 2.2.2 Setting

Through the Questionnaire Star platform, it was set that the same IP address and the same mobile phone number could only submit the questionnaire once. Compulsory questions, limited filling formats, and no blanks were set. Questionnaires with regular responses, filling times less than 120 s, and logical inconsistencies were eliminated. Quality control of the filled questionnaires was carried out at any time. The questionnaire was anonymous and did not involve private information or sensitive language. Participants gave informed consent and participated voluntarily to ensure the authenticity and reliability of the answer content.

#### 2.2.3 Data analyses

SPSS 26.0 software was used for data processing and analysis. Descriptive statistical analysis was used to analyze the general data of the respondents. Chi-square test was used to compare the count data between groups. If the data conformed to normal distribution and homogeneity of variance, two independent sample t-test was used to compare the means between the two groups. If the data did not conform to normal distribution, Mann–Whitney U test was used to compare the means between the two groups. Spearman correlation analysis was used to analyze the correlations between diet, exercise, quality of life and weight loss behavior. Binary Logistic regression analysis was used to analyze the influencing factors of weight loss. Variable selection was performed using the Enter method based on the results of Single factor analysis (*p* < 0.05). Statistical significance was set at *p* < 0.05. Collinearity was assessed by variance inflation factor (VIF), VIF < 5 as no severe collinearity was present. The goodness of fit of the model was evaluated using the Hosmer-Lemeshow test (with *p* > 0.05 indicating a good fit) and the Nagelkerke *R^2^*.

## 3 Results

### 3.1 Participants’ characteristics

A total of 362 electronic questionnaires were collected in this survey, of which 302 were valid questionnaires, with an effective recovery rate of 83.43%. The average age of college students was 22.15 ± 2.2 years, and the average body mass index (BMI) was 20.99 ± 2.92 kg/m^2^. Males accounted for 43.4% and females accounted for 56.6%. Most of the college students were 21–23 years (47.0%), normal weight (67.9%), Han nationality (95.0%), urban residence (65.9%), non-only child (71.2%), bachelor degree (58.6%), third grade (29.1%), medical students (53.6%), no medical practitioners in their family (68.9%), and parents with low education level; In terms of weight loss, the majority of college students did not have weight loss behavior (55.0%), moderate or weak weight loss intention (73.5%), and weight loss was influenced by social media (54.0%). See Table 1 for details.

**Table 1:** General characteristics of participants (*n* = 302).

Characteristics	Factors	Number characteristics	Proportion (%)
Gender	Male	131	43.4
	Female	171	56.6
Age	18–20	84	27.8
	21–23	142	47.0
	≥24	76	25.2
BMI	<18.5	57	18.9
	18.5– < 24.0	205	67.9
	≥24.0	40	13.2
Nation	Han nationality	287	95.0
	Ethnic minorities	15	5.0
Residence	City	199	65.9
	Rural area	103	34.1
Only child	Yes	87	28.8
	No	215	71.2
Academic level	Specialty	35	11.6
	Bachelor’s degree	177	58.6
	Master’s degree	90	29.8
Grade	First grade	52	17.2
	Second grade	83	27.5
	Third grade	88	29.1
	Fourth grade	63	20.9
	Fifth grade	16	5.3
Medical student	Yes	162	53.6
	No	140	46.4
Weight loss behavior	Yes	136	45.0
	No	166	55.0
Weight loss intention	Strong	80	26.5
	Moderate or weak	222	73.5
Influenced by social media	Yes	163	54.0
	No	139	46.0
Family medicine practitioner	Yes	94	31.1
	No	208	68.9
Father’s educational level	Primary school and below	27	8.9
	Junior high school	96	31.8
	High school	65	21.5
	Junior college	54	17.9
	Bachelor’s degree or above	60	19.9
Mother’s educational level	Primary school and below	54	17.9
	Junior high school	84	27.8
	High school	59	19.5
	Junior college	52	17.2
	Bachelor’s degree or above	53	17.5

### 3.2 Status of weight loss behavior and scores of diet, exercise and quality of life in college students

The survey found that 136 college students (45.0%) had weight loss behavior, including 63 male students and 73 female students. The total score of DEBQ was 101.41 ± 23.84, the total score of SEE-C was 38.55 ± 19.67, and the scores of each dimension of SF-36 were: PF was 85.20 ± 15.98, RP was 74.83 ± 34.88, BP was 72.66 ± 21.20, GH was 65.86 ± 18.08, VT was 62.20 ± 15.82, SF was 71.71 ± 20.30, RE was 52.10 ± 42.19, MH was 61.11 ± 15.75. The comparison between the reduced-weight and non-reduced-weight groups showed significant statistical differences in DEBQ and its various dimensions, as well as SEE-C, *p* < 0.05. See Table 2 for details.

**Table 2:** Comparison results of college students’ scores in diet, exercise and quality of life.

Projects	Overall	reduced-weight groups^①^	95% CI	t/U	*p*
		non-reduced-weight groups^②^			
DEBQ	101.41 ± 23.84	111.54 ± 22.91^①^	13.414–23.446	7.230	<0.001
		93.11 ± 21.30^②^			
Restrained eating	30.30 ± 9.12	35.88 ± 7.31^①^	8.416–11.876	11.539	<0.001
		25.73 ± 7.83^②^			
Emotional eating	36.32 ± 13.03	39.49 ± 12.91^①^	2.880–8.672	3.924	<0.001
		33.72 ± 12.60^②^			
External eating	34.80 ± 7.39	36.18 ± 6.63^①^	0.848–4.167	−3.085	0.002
		33.67 ± 7.79^②^			
SEE-C	38.55 ± 19.67	42.75 ± 19.26^①^	3.243–12.041	3.419	0.001
		35.11 ± 19.38^②^			
SF-36					
Physical function	85.20 ± 15.98	85.51 ± 16.48^①^	−3.067-4.217	−0.775	0.438
		84.94 ± 15.60^②^			
Role physical	74.83 ± 34.88	74.82 ± 33.68^①^	−7.986–7.919	−0.473	0.636
		74.85 ± 35.94^②^			
Bodily pain	72.66 ± 21.20	71.54 ± 21.84^①^	−6.864-2.793	−0.619	0.536
		73.57 ± 20.69^②^			
General health	65.86 ± 18.08	65.74 ± 18.27^①^	−4.350-3.893	−0.025	0.980
		65.96 ± 17.97^②^			
Vitality	62.20 ± 15.82	60.88 ± 16.64^①^	−5.997-1.195	−0.908	0.364
		63.28 ± 15.07^②^			
Social function	71.71 ± 20.30	70.18 ± 20.81^①^	−7.396-1.839	−1.110	0.267
		72.96 ± 19.85^②^			
Role emotion	52.10 ± 42.19	50.49 ± 40.97^①^	−12.537-6.690	−0.612	0.540
		53.41 ± 43.25^②^			
Mental health	61.11 ± 15.75	59.09 ± 16.24^①^	−7.249--0.117	−1.960	0.050
		62.77 ± 15.18^②^			

### 3.3 Single factor analysis of influencing factors of weight loss behavior

The analysis results showed that the weight loss behavior of college students was statistically significant in relation to BMI, weight loss intention, and the influence of social media, *p* < 0.001. See Table 3 for details.

**Table 3:** Single factor analysis of influencing factors of weight loss behavior.

Factors	Weight loss behavior [Case (%)]		*χ^2^*	*p*	95% CI
	Yes	No			
Gender			0.874	0.350	0.787–1.965
Male	63 (48.1%)	68 (51.9%)			
Female	73 (42.7%)	98 (57.3%)			
Age			1.520	0.468	0.461–0.480
18–20	39 (46.4%)	45 (53.6%)			
21–23	59 (41.5%)	83 (58.5%)			
≥24	38 (50.0%)	38 (50.0%)			
BMI			42.372	<0.001	0.000–0.010
<18.5	7 (12.3%)	50 (87.7%)			
18.5– < 24.0	98 (47.8%)	107 (52.2%)			
≥24.0	31 (77.5%)	9 (22.5%)			
Nation			0.162	0.688	0.431–3.581
Han nationality	130 (45.3%)	157 (54.7%)			
Ethnic minorities	6 (40.0%)	9 (60.0%)			
Residence			0.155	0.693	0.563–1.465
City	88 (44.2%)	111 (55.8%)			
Rural area	48 (46.6%)	55 (53.4%)			
Only child			0.519	0.471	0.729–1.980
Yes	42 (48.3%)	45 (51.7%)			
No	94 (43.7%)	121 (56.3%)			
Academic level			0.175	0.916	0.910–0.964
Specialty	15 (42.9%)	20 (57.1%)			
Bachelor’s degree	79 (44.6%)	98 (55.4%)			
Master’s degree	42 (46.7%)	48 (53.3%)			
Grade			1.814	0.770	0.735–0.828
First grade	25 (48.1%)	27 (51.9%)			
Second grade	41 (49.4%)	42 (50.6%)			
Third grade	36 (40.9%)	52 (59.1%)			
Fourth grade	28 (44.4%)	35 (55.6%)			
Fifth grade	6 (37.5%)	10 (62.5%)			
Medical student			0.499	0.480	0.747–1.858
Yes	76 (46.9%)	86 (53.1%)			
No	60 (42.9%)	80 (57.1%)			
Weight loss intention			79.289	<0.001	8.034–34.073
Strong	70 (87.5%)	10 (12.5%)			
Moderate or weak	66 (29.7%)	156 (70.3%)			
Influenced by social media			14.831	<0.001	1.559–3.985
Yes	90 (55.2%)	73 (44.8%)			
No	46 (33.1%)	93 (66.9%)			
Family medicine practitioner			0.007	0.934	0.600–1.599
Yes	42 (44.7%)	52 (55.3%)			
No	94 (45.2%)	114 (54.8%)			
Father’s educational level			2.762	0.598	0.573–0.648
Primary school and below	10 (37.0%)	17 (63.0%)			
Junior high school	39 (40.6%)	57 (59.4%)			
High school	30 (46.2%)	35 (53.8%)			
Junior college	28 (51.9%)	26 (48.1%)			
Bachelor’s degree or above	29 (48.3%)	31 (51.7%)			
Mother’s educational level			7.130	0.129	0.112–0.193
Primary school and below	16 (29.6%)	38 (70.4%)			
Junior high school	42 (50.0%)	42 (50.0%)			
High school	26 (44.1%)	33 (55.9%)			
Junior college	27 (51.9%)	25 (48.1%)			
Bachelor’s degree or above	25 (47.2%)	28 (52.8%)			

### 3.4 Correlation analysis of college students’ weight loss behavior with diet, exercise and quality of life

Spearman correlation analysis results showed that the weight loss behavior of college students was positively correlated with DEBQ and its various dimensions, as well as SEE-C, *p* < 0.05. See Table 4 for details.

**Table 4:** Correlation analysis of college students’ weight loss behavior with diet, exercise and quality of life.

Variables	Restrained eating	Emotional eating	External eating	DEBQ	SEE-C
Weight loss behavior	0.568**	0.217**	0.178**	0.398**	0.192**

**p < 0.01.

### 3.5 Logistic regression analysis of influencing factors of weight loss behavior

Taking the weight loss behavior of college students as the dependent variable and the variables with statistical significance in the single analysis (*p* < 0.05) as the independent variables, logistic regression analysis was conducted. Variables were assigned before the analysis to allow interpretation of the results. The collinearity diagnosis showed that the VIF of all independent variables were all less than 3, indicating the absence of collinearity. Hosmer-Lemeshow test *p* = 0.811 suggested that the model fit was good. Nagelkerke *R^2^* = 0.491 indicated that the model’s explanatory power was moderate. The results showed that BMI, weight loss intention, DEBQ and SEE-C were significantly correlated with weight loss behavior (*p* < 0.05). The probability of weight loss for college students with normal BMI and overweight was 3.462 and 15.912 times that of underweight, respectively. The probability of weight loss for college students with a strong weight loss intention was 8.566 times that of moderate or weak weight loss intention. For every 1-point increase in DEBQ and SEE-C, the weight loss behavior of college students increased by 1.031 and 1.023 times, respectively. *p* < 0.05. See Tables 5 and 6 for details.

**Table 5:** Independent variables assignment.

Variables	Assignment method
BMI	0 = <18.5; 1 = 18.5- < 24.0; 2 = ≥24.0
Weight loss intention	0 = Moderate or weak; 1 = Strong
Influenced by social media	0 = No; 1 = Yes
DEBQ	Substituting the original value
SEE-C	Substituting the original value

**Table 6:** Logistic regression analysis of influencing factors of weight loss behavior.

Factors	*β*	S. E.	Wald *χ^2^*-value	*p*	OR value	95% CI
Constant	−6.144	0.977	39.547	<0.001	0.002	
BMI						
<18.5 (reference)						
18.5- < 24	1.242	0.478	6.764	0.009	3.462	1.358–8.827
≥24.0	2.767	0.645	18.416	<0.001	15.912	4.496–56.309
Weight loss intention						
Moderate or weak (reference)						
Strong	2.148	0.395	29.629	<0.001	8.566	3.953–18.564
Influenced by social media						
No(reference)						
Yes	0.332	0.324	1.050	0.306	1.394	0.739–2.630
DEBQ	0.031	0.008	15.106	<0.001	1.031	1.015–1.047
SEE-C	0.023	0.008	7.128	0.008	1.023	1.006–1.040

## 4 Discussion

This study analyzed the weight loss behavior and influencing factors of 302 college students. The results showed that 45.0% of the college students had weight loss behavior. BMI, weight loss intention, DEBQ and SEE-C were significant related factors influencing the weight loss behavior of college students. Compared with the underweight group, the odds of overweight college students showing weight loss behavior was nearly 16 times higher, while that of normal BMI was 3.5 times higher. This likely reflected an expected behavior that overweight students were more likely to attempt to lose weight. BMI, as an objective indicator, was the most powerful factor in predicting weight loss behavior and the most direct source of perceived susceptibility. Perceiving susceptibility and severity were the prerequisite for changes in healthy behavior (20). Study showed that BMI was significantly positively correlated with perceived stress (21), indicating that college students who were overweight or had a normal upper weight might be more likely to perceive the physical changes or social evaluation stress brought about by weight, thereby triggering the motivation to lose weight. However, as this study was a cross-sectional design, the result only indicated a significant association between BMI and weight loss behavior, and could not be inferred causal relationship between the two. It could not be concluded that overweight directly led to weight loss behavior. It could only suggest that weight status was an important associated factor for weight loss behavior.

In terms of psychological motivation, the results highlighted the weight loss intention was the key psychological motivation and bridge role of behavior transformation. In this study, the odds of weight loss of people with strong willingness was 8.5 times higher than that of moderate or weak willingness. This was consistent with the research results of Zhang (22), indicating that weight loss intention was conducive to the implementation of weight loss behavior, and appropriately strengthening weight loss intention could help with weight loss (23). This suggested that general intention alone might not be enough to drive sustained behavior. In subsequent weight loss interventions, health education might be used to help college students correctly understand the health risks of being overweight and could transform their general weight loss desire into firm behavioral commitment, thereby strengthening the psychological driving force for weight loss.

DEBQ was positively correlated with weight loss behavior, and the odds of weight loss increased by 1.031 times for each increase of 1 point, reflecting the change of the measurement connotation of the scale in this population. This might be related to the active adoption of dietary control strategies by weight loss people. The scores in the restrained eating dimension might be higher, indicating the weight loss students could more consciously control their food intake and avoid high-calorie foods, rather than emotional eating. In this context, it more prominently reflected the efforts of active dietary management. Research showed that restrained eating score was significantly associated with physical dissatisfaction (24). The negative body image generated by college students’ dissatisfaction with their body shape might transform into strong expectation of idealized slender figure. The gap between the negative perception of body shape and the positive ideal image could effectively stimulate the behavior tendency of diet control, which might be beneficial for overweight or obese young adults to lose weight and subsequently lower their BMI (25), promoting the initiation and maintenance of weight loss behavior, and ultimately had positive impact on the weight loss effect (26). Meanwhile, this result could either be due to the active dietary control tendency promoting the implementation of weight loss behavior, or it might be that after participating in weight loss behavior, the dietary control ability of college students improved, resulting in an increase in the score of DEBQ. The dynamic correlation between these two factors could need to be further verified through longitudinal studies in the future.

SEE-C was positively correlated with weight loss behavior. For every 1-point increase, the odds of weight loss increased by 1.023 times, indicating that higher sense of exercise self-efficacy might have a certain promoting effect on weight loss, which was consistent with the research results of Albasheer et al. (27) Self-efficacy was an important component in reducing the risk of obesity among college students (28), reflecting their confidence in their ability to perform healthy behaviors. People with higher self-confidence were more likely to adopt healthy behaviors to improve their health (20), thereby promoting weight loss behavior. College students with high self-efficacy were better able to overcome psychological issues such as procrastination and fear, actively engaged in physical activities and devoted their energy to regular exercise. They might also be more adept at finding solutions to exercise obstacles, thereby being more likely to initiate and maintain weight loss behavior, and promoting the implementation of weight loss.

This study adopted the convenience sampling method. The proportion of medical students reached 53.6%. Due to their professional background, medical students had stronger health awareness and knowledge related to weight loss. Their weight loss behavior characteristics might differ from those of non-medical students. The sample had certain selection bias and self-selection bias. The conclusion was only applicable to the college students within the sampling range of this study and could not be directly generalized to all Chinese college students. Further research might need to expand the sample representativeness through stratified random sampling. Furthermore, this study found that the influence of social media was of significant importance in the single factor analysis. Although it might be masked by more direct individual factors in the multi-factor model, its significance could not be ignored. This was a discovery with distinct characteristics of the times, suggesting that social media might provide external stimulation and community support for college students to lose weight by shaping body image standards, disseminating fitness and weight loss knowledge, and building online fitness communities (29), which was an important external factor for stimulating weight loss motivation. Subsequent interventions could make full use of this carrier to create positive weight loss atmosphere.

This study found that objective physiological needs were the original motivation for weight loss behavior, and strong weight loss intention was the decisive psychological state to transform motivation into action plan. Driven by this, positive dietary control behaviors and high confidence in exercise jointly constituted the guarantee at the execution level. The results of this study would have certain practical guiding significance for universities in conducting weight management for college students, providing precise and operational intervention strategies. Firstly, for overweight and critical weight students, universities should shift the focus of health education from the dissemination of health knowledge to enhancing their perceived susceptibility and severity of health risks related to their own weight. Through methods such as body index interpretation and visualization of health risks, the objective BMI data could be transformed into individualized health warnings, stimulating their intrinsic motivation to lose weight. Secondly, regarding students’ weight loss intention, efforts should be made to enhance the cultivation of their motivation for weight reduction and facilitate the transformation of this desire into actual actions. Universities should utilize various channels such as campus lectures, public accounts, and bulletin boards to educate college students about the dangers of overweight and obesity, share successful weight loss stories, set short-term goals, etc. helping them convert the vague desire to lose weight into specific and firm commitments to act, stimulating their motivation for weight loss and guiding them to establish scientific concept of weight management, promoting the transformation from desire to action. Thirdly, regarding students’ dietary behaviors, universities should offer public elective courses or lectures related to healthy eating and nutrition combination, as well as practical courses such as cafeteria ordering guide. Nutrition professional teachers and medical staff should be invited to teach college students scientific diet control methods, such as high-calorie food identification, intake control, reasonable matching of three meals, etc., to improve their actual ability of diet management, not only nutrition theory infusion. Fourthly, when it comes to students’ self-efficacy in sports, the principle of gradual progress should be followed. Universities should offer diverse, engaging, and low-entry-level sports activities (such as yoga, campus challenges, and fun sports events), improve campus sports facilities, increase sports venues and equipment, meet the sports needs of different students, teach sports skills, help students build confidence through successful experiences, and gradually cultivate regular exercise habits, thereby enhancing the sports ability of college students. For traditional Chinese medicine (TCM) colleges, based on the unique characteristics of the TCM major, they could carry out weight management interventions with TCM features, such as traditional Chinese dietary therapy, acupoint massage, Baduanjin and Tai Chi providing diverse weight loss methods for college students. In the era of rapid development of information networks, it might be also necessary to be adept at making use of social media such as Douyin and Xiaohongshu to disseminate scientific knowledge about weight loss and create positive and scientific online health communities. By sharing successful experiences, providing exemplary demonstrations, and offering positive feedback, supportive environment could be created through peer effects and the power of role models.

## 5 Strengths and limitations

This study was the first to explore the relationship among dietary behavior, exercise self-efficacy and weight loss behavior among college students majoring in TCM. It revealed that BMI, weight loss intention, dietary behavior and exercise self-efficacy were the core influencing factors of weight loss behavior in this group. This result provided precise target direction for weight management intervention for college students. At the same time, it clarified the intensity and mechanism of each factor’s effect on weight loss behavior, providing scientific basis for subsequent targeted health intervention. However, this study had certain limitations. This study adopted a cross-sectional design and could not infer causal relationships among variables. For instance, the positive correlation between dietary behavior scores and weight loss behavior could not be distinguished. It might be impossible to determine whether active dietary control promoted weight loss or whether weight loss behavior enhanced the ability to control diet. Moreover, it was also unable to track the long-term maintenance effect of weight loss behavior. The sample was recruited through social media, and the proportion of medical students was relatively high. The respondents came from only one province and one university, which limited the representativeness of the sample. There might lead to selection bias and self-selection bias. The conclusion was only applicable to the group of college students within the sampling scope of this study. Dietary patterns and exercise self-efficacy all relied on self-administered scales by students and might be influenced by recall bias or subjective beautification, especially for sensitive topics such as weight and diet. The research subjects might have provided embellished answers regarding these issues. Although psychological, behavioral, and social variables were included in the study, some potentially important factors, such as sleep quality and stress level, were omitted. Future research could adopt longitudinal design to track the entire process from the generation of intention to the maintenance of behavior, in order to clarify the causal paths and dynamic interactions among various factors. The bias from retrospective data could be reduced by adopting some more objective, continuous and contextualized measurements (such as dietary diaries) of health behaviors. Expanding the source of the sample to cover students from different regions, different types of universities and majors to enhance representativeness of the research conclusions could provide empirical evidence for formulating scientific and feasible weight management plans for college students.

## 6 Conclusion

BMI, weight loss intention, dietary behavior and exercise self-efficacy were the influencing factors of weight loss behavior among college students. Overweight or obese can cause serious harm to physical and mental health. College students should enhance their health awareness, have reasonable diet voluntarily, strengthen physical exercise, improve their self-efficacy in sports, and develop good habits of scientific exercise and diet. Colleges and universities should also do good job in publicity and guidance, strengthen health education and behavioral intervention, formulate practical and feasible health promotion strategies based on the actual situation of students, actively guide college students to establish good lifestyle, urge students to strengthen self-health management, improve students’ self-management ability, and maintain healthy weight and body.

## Data Availability

The raw data supporting the conclusions of this article will be made available by the authors, without undue reservation.
